# Granulomatosis with polyangiitis mimicking COVID‐19 pneumonia: A case report

**DOI:** 10.1002/ccr3.8007

**Published:** 2023-10-06

**Authors:** Arjun Basnet, Sajog Kansakar, Nava Raj Sharma, Sudarshan Gautam, Saral Lamichhane, Kripa Tiwari, Madalasa Pokhrel, Sehajpreet Singh

**Affiliations:** ^1^ Maimonides Medical Center Brooklyn New York United States; ^2^ Manipal College of Medical Sciences Pokhara Nepal; ^3^ Gandaki Medical College Pokhara Nepal; ^4^ Montefiore New Rochelle Hospital New Rochelle New York United States

**Keywords:** ANCA‐associated vasculitis, COVID‐19, granulomatosis with polyangiitis

## Abstract

Granulomatosis with polyangiitis (GPA), formerly known as Wegener's granulomatosis, is a necrotizing vasculitis characterized by small‐to‐medium‐sized vessel involvement and the presence of antineutrophil cytoplasmic antibodies (ANCA). We present a case of a 26‐year‐old Asian woman who was transferred to our center from a nearby hospital, where she presented with shortness of breath, tested positive for COVID‐19, and was being managed for COVID‐19 pneumonia. She also had hemoptysis, skin lesions, and left foot numbness. Serological markers and VATS‐guided lung biopsy confirmed the diagnosis. Treatment with methylprednisolone and rituximab led to stabilization, despite complications of subcutaneous emphysema and lower extremity neuropathic symptoms. Early recognition and appropriate management of GPA are crucial for optimal outcomes.

## Graphical abstract

In this unique case report, we present a 26‐year‐old Asian female initially diagnosed with COVID‐19 pneumonia who was ultimately found to have Granulomatosis with Polyangiitis (GPA). Despite her young age and COVID‐19‐like symptoms, further evaluation, including serological markers and VATS‐guided lung biopsy, confirmed the GPA diagnosis. Treatment with methylprednisolone and rituximab led to stabilization, emphasizing the importance of considering GPA in the differential diagnosis, even in younger individuals presenting with respiratory symptoms suggestive of COVID‐19. Early recognition and appropriate management of GPA are crucial for optimal outcomes.

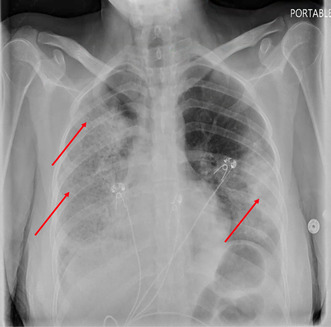

## 1 INTRODUCTION

Granulomatosis with Polyangiitis (GPA) is a small‐medium vessel necrotizing vasculitis and is a component of antineutrophil cytoplasmic antibody (ANCA)‐associated vasculitides.^1^ It has a peak incidence at 64–75 years of age and is commonly reported in Caucasians without sex predilection.^2^ Although almost any organ can be involved in GPA, the upper respiratory tract, lower respiratory tract, and kidneys are commonly affected.^3^ The severity of the disease can be heterogeneous. We report a unique case of GPA in a 26‐year‐old Asian woman who was initially transferred from an outside hospital due to shortness of breath presumed to be caused by COVID‐19 pneumonia. However, further evaluation led to the diagnosis of GPA highlighting the importance of considering GPA in the differential diagnosis, even in younger individuals and those presenting with respiratory symptoms suggestive of COVID‐19.

## 2 CASE PRESENTATION

A 26‐year‐old Asian woman with a medical history of migraine was transferred to our center from a nearby hospital, where she presented with shortness of breath, tested positive for COVID‐19, and was being managed for COVID‐19 pneumonia. In the outside center, she was being managed with supplemental oxygen, remdesivir, dexamethasone, and levofloxacin to cover community‐acquired pneumonia (CAP). During the hospitalization, she developed hemoptysis. The tuberculosis workup was negative. She was started on baricitinib and transferred to our center for specialized care and further workup, given worsening symptoms despite standard treatment.

At the presentation to our center, she complained of chest pain. A review of systems was notable for paresthesia on the dorsal aspect of the left foot. She was tachycardic and required a high‐flow nasal cannula to maintain oxygen saturation >92%. Physical examination revealed diffuse bilateral crackles on lung auscultation and nodular non‐blanching violaceous skin lesions on bilateral legs, which she attributed to shaving her legs. A patchy area of numbness was appreciated on the dorsal surface of the left leg. Initial laboratory results were significant for leukocytosis, elevated lactate dehydrogenase (LDH), procalcitonin, erythrocyte sedimentation rate (ESR), and C‐reactive protein (CRP), as summarized in Table 1. Severe acute respiratory syndrome coronavirus 2 (SARS‐CoV‐2) was detected on polymerase chain reaction (PCR). Electrocardiogram (EKG) revealed sinus tachycardia.

**TABLE 1:** Initial laboratory values.

Tests	Result	Reference range
Sodium, serum	137 mmol/L	135–149 mmol/L
Potassium, serum	4.5 mmol/L	3.4–4.8 mmol/L
Chloride, serum	99 mmol/L	93–105 mmol/L
Bicarbonate, serum	27 mmol/L	23–32 mmol/L
Blood urea nitrogen, serum	16.0 mg/dL	7–21 mg/dL
Creatinine, serum	0.6 mg/dL	0.3–1.1 mg/dL
White blood count	10.4 k/μL	4.8–10.8 k/μL
Hemoglobin	10.5 g/dL	12–16 g/dL
Platelets	512 k/μL	150–450 k/μL
Lactate dehydrogenase	482 IU/L	108–199 IU/L
C‐reactive protein	13.624 mg/dL	0.0–0.9 mg/dL
Erythrocyte sedimentation rate	45 mm/h	0–20 mm/h

**FIGURE 1:**
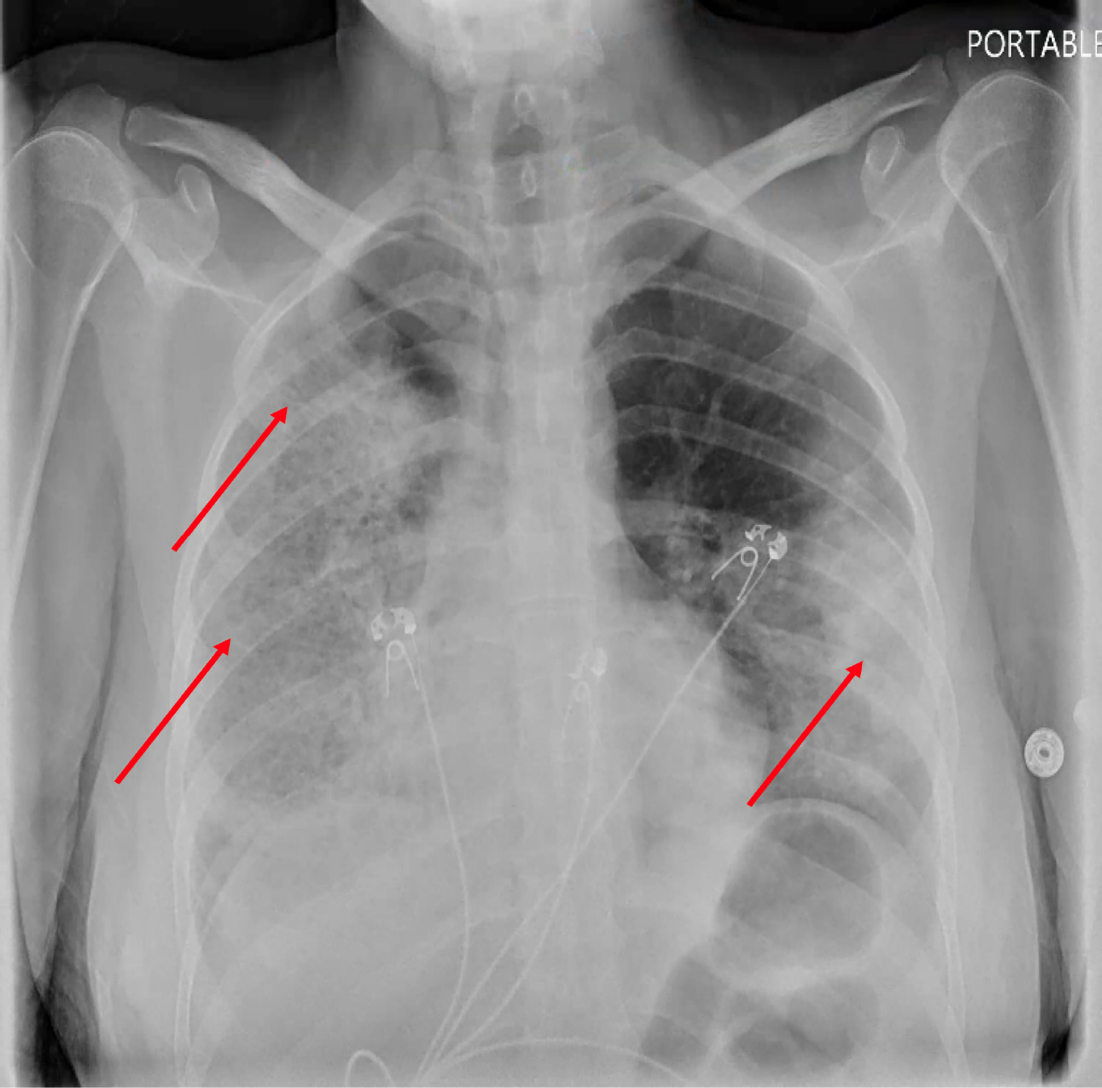
Chest X‐ray showing extensive right lung opacity and left mid to lower lung opacity as shown by the arrows.

**FIGURE 2:**
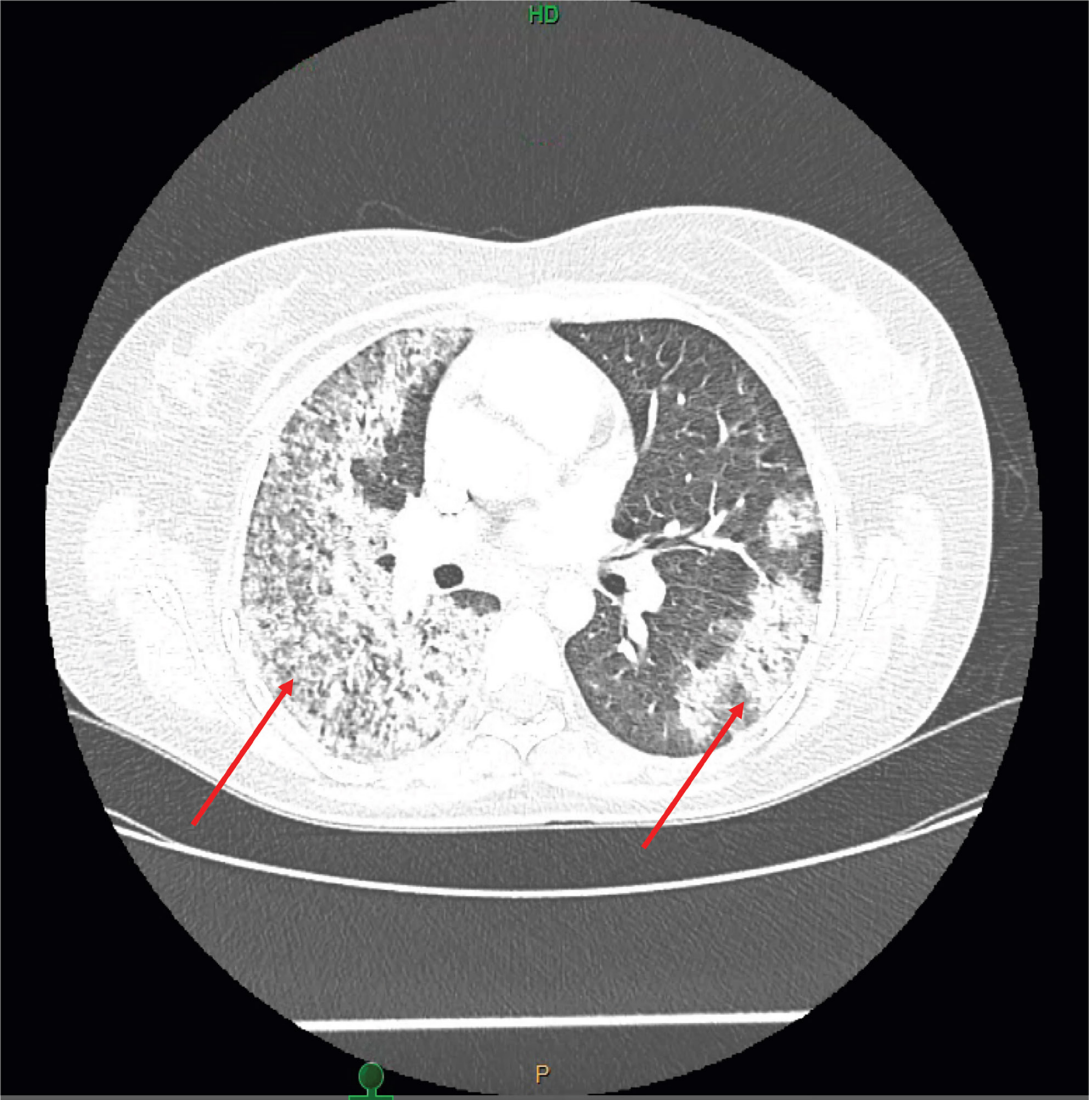
Computed tomography (CT) chest showing dense consolidation of the entire right lung and peripheral infiltrates in the left lung.

The patient's urinalysis showed trace protein, moderate hemoglobin, and urine red blood cell (RBC) count of 25–50 per high power field (HPF). Chest X‐ray (CXR) revealed extensive opacity in the right lung and mid‐to‐lower lung opacity on the left as shown in Figure 1. Computed tomography (CT) chest showed dense consolidation in the entire right lung and peripheral infiltrates in the left lung as shown in Figure 2. The patient was admitted to the intensive care unit for COVID‐19 treatment. Rheumatology consultation was sought due to hemoptysis, skin lesions, and numbness in the left foot. Laboratory tests revealed an elevated rheumatoid factor, positive cytoplasmic C‐ANCA antibody, high C‐ANCA titer, and elevated PR‐3 antibody, raising suspicion of granulomatosis with polyangiitis (GPA) rather than COVID‐19 pneumonia with associated neuropathy and rash.

**FIGURE 3:**
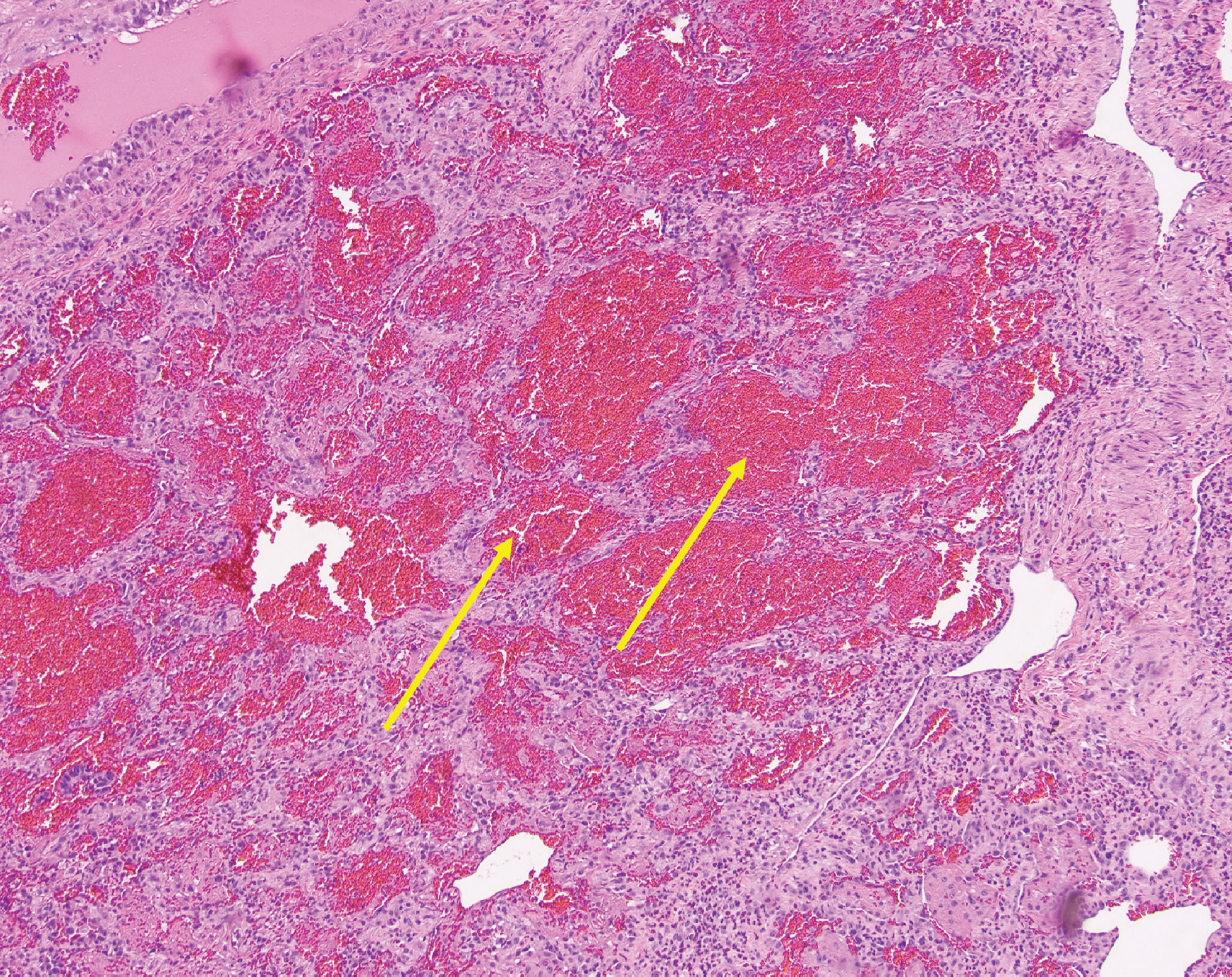
Video‐assisted thoracoscopic surgery (VATS)‐guided biopsy showing diffuse alveolar hemorrhage.

**FIGURE 4:**
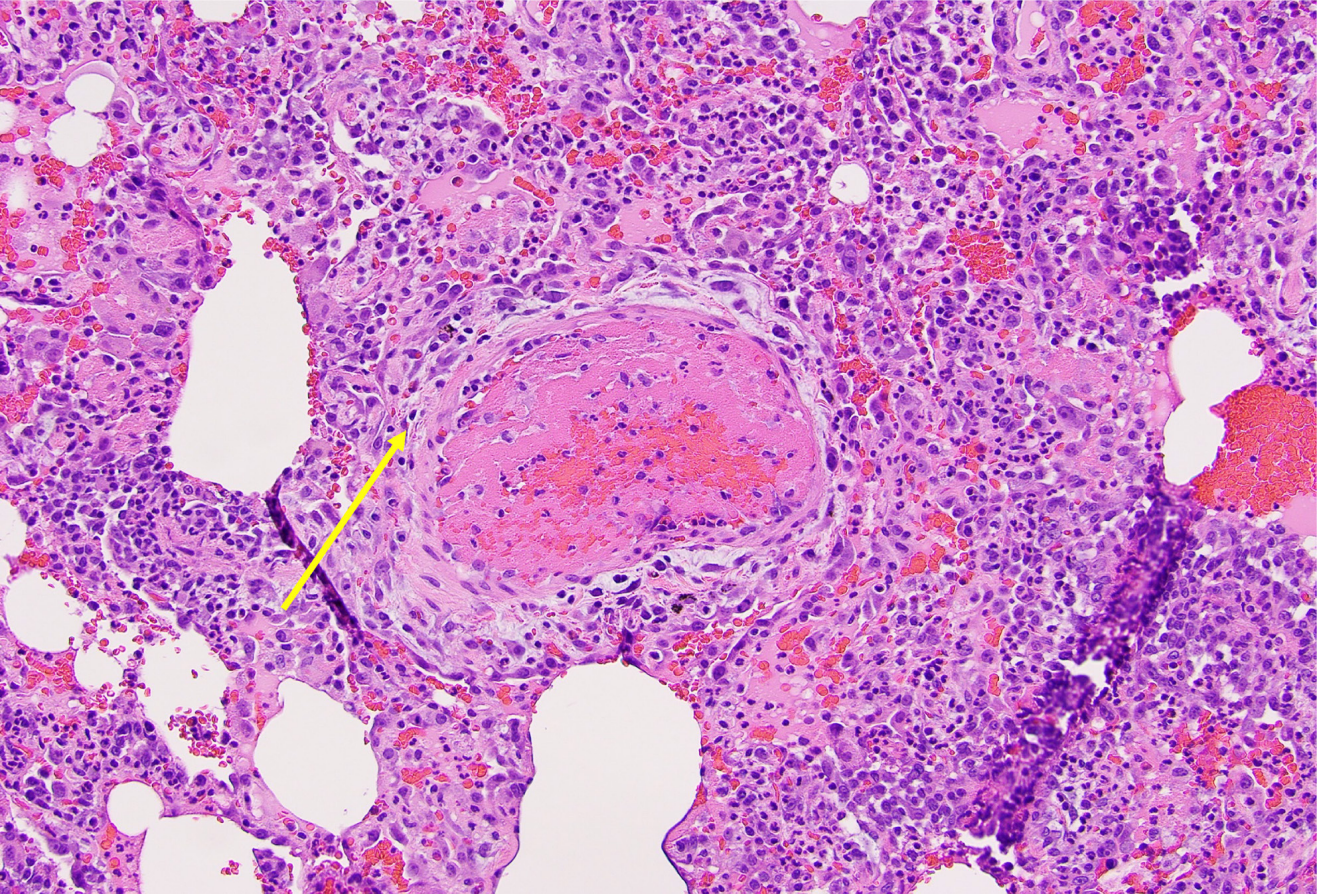
Video‐assisted thoracoscopic surgery (VATS)‐guided lung biopsy showing necrotizing vasculitis with fibrin and blood filling the alveolar space, inflammatory infiltration with neutrophils, scattered areas of necrosis, and endothelial damage.

To confirm the diagnosis, a video‐assisted thoracoscopic surgery (VATS)‐guided lung biopsy was performed, showing characteristic histological features of ANCA‐associated vasculitis, including fibrin and blood filling the alveolar space, inflammatory infiltrate with neutrophils, scattered areas of necrosis, endothelial damage, and necrotizing vasculitis as shown in Figures 3 and 4. Despite the absence of well‐formed granulomata, the findings of necrotizing vasculitis with the area of endothelial damage and inflammatory infiltrate with neutrophils supported the diagnosis of GPA. Treatment with methylprednisolone and rituximab led to medical stabilization, although the patient experienced complications of subcutaneous emphysema and progressive lower extremity neuropathic symptoms.

Following successful stabilization, the patient was discharged home with a walker due to a foot drop and was scheduled for outpatient neurology and rheumatology follow‐up.

## 3 DISCUSSION

GPA, previously known as Wegener's granulomatosis, is the most common pulmonary vasculitis and is associated with ANCA to proteinase 4 (PR3). Microscopic polyangiitis (MPA) and eosinophilic granulomatosis, and polyangiitis (Churg Strauss syndrome) are also associated with ANCAs, often against myeloperoxidase (MPO), and are grouped within ANCA‐associated vasculitis.^4^ GPA is a rare disease with an estimated incidence of 0.4–11.9 cases per 1 million person‐years predominantly affecting populations of European descent, while MPA predominates in Asian populations.^5^ The typical age of onset is 45–65 years with no sex predilection. Our patient was a 26‐year‐old Asian woman, demonstrating that any age group and ethnicity might be affected.

GPA is heterogeneous in its spectrum and severity of presentation. Up to 80% of patients present with prodromal clinical features of systemic inflammation, such as fever, weight loss, fatigue, myalgia, and arthralgia.^4^ Rarely, patients may present more acutely over days. It commonly affects the upper respiratory tract (e.g., sinuses, nose, ears, pharynx, and trachea), lower respiratory tract, and kidneys. A varying degree of disseminated vasculitis can occur that can affect any organ. Pulmonary involvement is seen in 50%–90% of cases.^6^ It can manifest as cough, dyspnea, and a wide variety of imaging abnormalities such as single or multiple nodules, cavitation, ground‐glass opacities, consolidation (reflecting inflammation or alveolar hemorrhage), effusions, stenosis of trachea and bronchi, and rarely fibrosis.^6^, ^7^ Glomerulonephritis occurs in 70%–85% of patients, with the characteristic lesion being a segmental focal glomerulonephritis, with rapidly progressive glomerulonephritis (RPGN) seen in fulminant cases.^4^ The onset and course are variable, and ESRD develops in 11%–32% of the patients.^8^

Our patient developed a sudden onset of respiratory symptoms without any prodrome, which is uncommon. Imaging revealed dense consolidation of the entire right lung and peripheral infiltrates in the left lung, and she tested positive for SARS‐CoV‐2. This clinical picture resembled COVID‐19 pneumonia very closely. No upper respiratory tract involvement was seen, and microscopic hematuria was seen, which is non‐specific. As a result, she was initially managed with therapy directed toward COVID‐19 pneumonia. The lack of improvement with COVID‐19‐directed treatment, the nodular non‐blanching violaceous skin lesions on bilateral legs, and left foot numbness (that eventually progressed to distal sciatic neuropathy) raised suspicion for GPA in this case. Skin involvement can present as palpable purpura, nodules, ulcers, and maculopapular rashes and can correlate with active disease. A biopsy can demonstrate leukocytoclastic vasculitis or granulomatous vasculitis, which are not specific to GPA.^9^ Nervous system involvement is less common but presents most commonly as mononeuritis multiplex, as seen in our case. Other reported manifestations to include cerebral infarction or bleeding, seizures, cranial nerve palsies, altered mental status, meningismus, quadriparesis, or paraparesis.^10^, ^11^

A biopsy was pursued to confirm the diagnosis in our case. A VATS‐guided biopsy of the lung was performed rather than a kidney biopsy, as the yield was likely to be low in the presence of only microscopic hematuria. Histopathological features of GPA described in the literature include necrotizing vasculitis involving venules, arterioles, and capillaries, granulomatous inflammation with/without parenchymal necrosis, micro‐abscesses, and fibrosis.^4^ Histopathological examination of the lung specimen of our patient revealed alveolar hemorrhage, micro‐abscesses, and necrotizing vasculitis consistent with GPA, even in the absence of granulomas.

Treatment of GPA is divided into the induction phase, to achieve remission in 3 months, and the maintenance phase, to maintain remission. Glucocorticoids, along with either cyclophosphamide or rituximab, are the standard of care for inducing remission in severe diseases, such as in our case.^12^ Rituximab is becoming preferred with growing evidence supporting its efficacy and benefits, such as fertility preservation, superiority in PR3‐ANCA positive patients, and relapsing disease.^5^ Thus, rituximab was preferred over cyclophosphamide in our patient with subsequent stabilization of her condition over several days. The systemic nature of ANCA‐associated vasculitis necessitates a multidisciplinary approach for diagnosis and management. Neurologists play a pivotal role in addressing the disruptive neuropathic symptoms that significantly affect patients' quality of life even after the induction of remission.^13^

## 4 CONCLUSION

This case emphasizes the importance of considering GPA in the differential diagnosis, particularly in younger patients with respiratory symptoms. Early recognition and accurate diagnosis are crucial for appropriate management and improved outcomes in GPA. Further studies are needed to explore the potential association between GPA and COVID‐19, as well as to better understand the heterogeneous nature of the disease.

## AUTHOR CONTRIBUTIONS

**Arjun Basnet:** Conceptualization; methodology; writing – original draft. **Sajog Kansakar:** Conceptualization; methodology; writing – original draft. **Nava Raj Sharma:** Conceptualization; methodology; writing – original draft; writing – review and editing. **Sudarshan Gautam:** Conceptualization; methodology; writing – original draft. **Saral Lamichhane:** Conceptualization; methodology; writing – original draft; writing – review and editing. **Kripa Tiwari:** Conceptualization; methodology; writing – original draft. **Madalasa Pokhrel:** Conceptualization; methodology; writing – original draft. **Sehajpreet Singh:** Conceptualization; methodology; writing – original draft; writing – review and editing.

## FUNDING INFORMATION

None.

## CONFLICT OF INTEREST STATEMENT

None.

## CONSENT

Written informed consent was obtained from the patient to publish this report in accordance with the journal's patient consent policy.

## Data Availability

Data is available on request.
